# An Optimized Structure-Function Design Principle Underlies Efficient Signaling Dynamics in Neurons

**DOI:** 10.1038/s41598-018-28527-2

**Published:** 2018-07-11

**Authors:** Francesca Puppo, Vivek George, Gabriel A. Silva

**Affiliations:** 1 Department of Bioengineering, University of California, San Diego, La Jolla, 92093 CA USA; 2 Department of Neurosciences, University of California, San Diego, La Jolla, 92093 CA USA; 3 Center for Engineered Natural Intelligence, University of California, San Diego, La Jolla, 92093 CA USA

## Abstract

Dynamic signaling on branching axons is critical for rapid and efficient communication between neurons in the brain. Efficient signaling in axon arbors depends on a trade-off between the time it takes action potentials to reach synaptic terminals (temporal cost) and the amount of cellular material associated with the wiring path length of the neuron’s morphology (material cost). However, where the balance between structural and dynamical considerations for achieving signaling efficiency is, and the design principle that neurons optimize to preserve this balance, is still elusive. In this work, we introduce a novel analysis that compares morphology and signaling dynamics in axonal networks to address this open problem. We show that in Basket cell neurons the design principle being optimized is the ratio between the refractory period of the membrane, and action potential latencies between the initial segment and the synaptic terminals. Our results suggest that the convoluted paths taken by axons reflect a design compensation by the neuron to slow down signaling latencies in order to optimize this ratio. Deviations in this ratio may result in a breakdown of signaling efficiency in the cell. These results pave the way to new approaches for investigating more complex neurophysiological phenomena that involve considerations of neuronal structure-function relationships.

## Introduction

The mechanisms underlying the successful integration and rapid transmission of information in the brain rely on interactions between structural and dynamical properties that begin at the level of the single neuron. The complexity of these interactions are reflected in the wide variability of axon arbor morphologies and dynamical states neurons can take on. A still unsolved fundamental question is what is the relationship between the morphological design principles of individual branching axons and their role in optimizing action potential signaling in the neuron?

Neuronal morphologies are the outcome of complex developmental processes including axon growth, stabilization of synaptic connections and axon pruning^1–3^. These processes are dependent on a multitude of local molecular and cellular mechanisms and conditions^4–6^. Despite the stochastic nature of morphological development, as well as other biological, physical, and molecular constraints, evolutionarily neurons have achieved a degree of common computational efficiency. A growing list of experimental and computational results, including those we present in this paper, suggest that these developmental processes in neurons satisfy a set of specific optimization principles^7–10^.

Until recently, the prevailing dominant hypotheses has been that neurons are morphologically designed to optimize for the least amount of cellular material necessary. The logic was that the less amount of material that was used, the greater the conservation of energy and cellular resources. In particular, a number of studies have argued that wiring minimization principles that maximize the conservation of material underlie the morphological design of neurons and even the broader anatomical organization responsible for functional maps in the neocortex^7,10–13^, such as the intracortical wiring underlying functional maps in mammalian visual cortex^10,14^. However, more recent work has shown that neurons are not minimized for wiring length but instead are designed somewhere in between the two extremes of minimizing wiring costs versus maximizing action potential conduction velocities. They use more material than minimal construction costs would allow in order to increase conduction velocities that decrease temporal costs, but at the same time they do not signal as fast at they could if the wiring design was optimized strictly for speed, thereby offsetting the material cost^9,15^. Budd *et al*. have recently examined the functional consequences and temporal cost of wiring minimization principles. Their results suggest that there exists a trade-off between the time it takes action potentials to reach synaptic terminals (temporal cost) and the amount of cellular material associated with the wiring path length of the neuron’s morphology (material cost)^9^. This can be interpreted as a need to balance the advantages to the organism of signaling and processing information as fast as possible, while keeping material and cellular costs within reason.

However, where this spatio-temporal trade-off lies and the design principles being optimized remain unknown. Identifying them is critical to ultimately understanding why neurons are designed the way they are, and the effect they may have on a neuron’s ability to represent and process information in the brain. This would also allow many other seemingly unrelated neurobiological results that involve considerations of neuronal structure-function relationships to be understood within this new context. As a result, completely disconnected results, models, and interpretations of data would have an underlying ‘constraint’ that nature necessitates they conform to. This would have a significant impact on our understanding of the brain as a system.

The work by Budd and colleagues suggested that the spatio-temporal trade-off observed in axon arbors contributes to the maximization of temporal precision, or conversely, the minimization of temporal dispersion in neuronal circuits^9^. Here, we explored the functional role of axon morphologies in the propagation of action potentials. The result of our analysis suggests a new putative underlying optimization principle between temporal and material costs in axon arbors. We investigated the signaling dynamics of action potentials in individual axonal branches using a model that computes a metric associated with the efficiency of signaling in spatial geometric networks. We have recently shown that optimal efficient signaling between connected node pairs in geometric networks is bounded by a ratio that approaches unity, between the signaling latency on the edge and the internal dynamics of the individual nodes^16^. We call this ratio the refraction ratio. It reflects a necessary balance between the internal dynamics of the participating nodes that make up the network, and the dynamics of signaling or information flow on the network. We have previously shown in numerical simulation experiments that a deviation of this principle can result in the complete breakdown of signaling dynamics^17^.

In this work, we propose the refraction ratio as a measure of signaling efficiency in axons, and we provide evidence that the design of axonal arborizations are constructed to optimize this ratio. We considered individual Basket cell neurons as geometric axon tree networks with nodes along axonal branching points. We then analyzed the signaling dynamics along individual axonal branches, where a branch is the entire axonal segment connecting the axon initial segment to one of the axon’s terminals. Basket cell neurons optimize the ratio between the refractory period of the membrane and action potential conduction latencies. Given a signaling latency along the convoluted path resulting from the morphological geometry of an axon arbor, the ratio of the signaling latency to the membrane refractory period approaches a value of unity for each individual axonal branch. This reflects the theoretically predicted optimal ratio.

One interpretation of our results is that the convoluted paths taken by axons and axonal branches reflect a design compensation by the neuron to slow down signaling latencies in order to optimize the refraction ratio. Because the cell does not have direct control over the biophysics responsible for determining the length and changing character of the membrane refractory period, it uses axonal morphology (structure) to ensure that an optimized refraction ratio is almost always preserved by the time action potentials reach synaptic terminals. An optimized ratio reflects a balance between the temporal constraints associated with the biophysics of the membrane, and the amount of time it takes action potentials (the signals) to travel down the axonal branch.

## Competitive Refractory Dynamics Model

We have recently described the construction and theoretical analysis of a framework derived from the canonical neurophysiological principles of spatial and temporal summation that models the competing dynamics of incident signals into nodes along directed edges in a network^16^. We considered how temporal latencies produce offsets in the timing of the summation of incoming discrete events, and the ultimate activation of downstream nodes. Our approach involved the development of a competitive refractory framework. The framework models how the timing of different signals compete to ‘activate’ nodes they connect into. We use the term activation here to imply an appropriate reaction of that node to the signal it has received. For example, in the case of biological neurons it would be the generation of an action potential that propagates down the axon and axonal arborizations. In an abstract notion of a directed network, it is the generation of a discrete signaling event by the activated node for an excitatory input (or lack of activation for an inhibitory input) and a subsequent period of refractoriness. The interplay between temporal latencies of propagating discrete signaling events on the network relative to the internal dynamics of the individual nodes can have profound effects on the dynamics of the network^17–20^. In a geometric network temporal latencies are due to the relationship between signaling speeds (conduction velocities) and the geometry of the edges on the network (i.e. edge path lengths). The model also assumes that each node has associated with it a refractory period or refractory state. The refractory period is a value that reflects the internal state of the node in response to its activation by an upstream winning node (or set of nodes). It is a period of time during which the node is not capable of responding to other input signals from nodes that connect into it. In other words, it is refractory to such inputs. We did not assume anything about the internal model that produces this refractory state, which could include an internal processing time during which the node is making a decision about how to react.

### The refraction ratio

We refer the reader to^16^ for a full treatment of the derivations and theoretical analysis. We considered the geometrical construction of a network in the following sense. We assumed that signals or discrete information events (e.g. action potentials in biological neurons) propagate between nodes along directed edges at a finite speed or conduction velocity, resulting in a temporal delay or latency of the signal arriving at the downstream node. Imposing the existence of signaling latencies implies a network that can be mapped to a geometric construction, where individual nodes could be assigned a spatial position in space in ℝ^3^. Directed edges connecting node pairs are allowed to have a convoluted path integral, i.e. a Jordan arc. There is no restriction that edges have to be spatially minimizing straight line edges, geodesics. A signaling latency *τ*_*ij*_ defines the ratio between the distance traveled on the edge relative to the speed of the propagating signal. For any set of connected vertex pairs *v*_*i*_*v*_*j*_, *τ*_*ij*_ = *d*_*ij*_/*s*_*ij*_. We then define a refractory period for the vertex *v*_*j*_ by *R*_*j*_. This reflects the internal dynamics of *v*_*j*_ once a signaling event activates it. For example, the amount of time the internal dynamics of *v*_*j*_ requires to make a decision about an output in response to being activated, or some reset period during which it cannot respond to subsequent arriving input signals. We place no restrictions on the internal processes or time that contribute toward *R*_*j*_. Once the winning node *v*_*j*_ ‘activates’, it will become refractory for a period of time *R*_*j*_. We can then compute at discrete times in parallel for every vertex in the network, i.e. every *v*_*j*_, which set of nodes that connects into *v*_*j*_ causally activates it. We are able to achieve this by keeping track of the temporal interplay of propagating signaling events on the edges relative to the refractory states of the individual vertices.

A key consideration of this computation was establishing a relationship between *R*_*j*_ and *τ*_*ij*_ for a vertex *v*_*j*_. We defined the refraction ratio between the refractory period *R*_*j*_ and a signaling latency *τ*_*ij*_ associated with a discrete signaling event into *v*_*j*_ coming from a vertex *v*_*i*_ on the edge *e*_*ij*_ as

1 $${{\rm{\Delta }}}_{ij}=\frac{{R}_{j}}{{\tau }_{ij}}=\frac{{R}_{j}\cdot {s}_{ij}}{{d}_{ij}}$$

where *R*_*j*_ > 0. Our determination of which set of *v*_*i*_ will successfully activate *v*_*j*_ emerges from an analysis of this ratio for every vertex connected into *v*_*j*_.

Under realistic conditions, there is likely to be a temporal offset between when each *v*_*i*_ signals and how far along *v*_*j*_ is in its recovery from its refractory period due to a previous signaling event. Furthermore, each contributing *v*_*i*_ is statistically independent from every other *v*_*i*_, so that the amount of temporal offset for each *v*_*i*_ vertex signaling *v*_*j*_ will be different. In order to compute these offsets and keep track of the overall dynamics of the network, we index two different notions of time. We defined *t*_*i*_ to be the moment at which *v*_*i*_ initiates a signal along its edge *e*_*ij*_ towards *v*_*j*_. And an observation time *T*_*o*_, which is the moment at which we observe or measure the state of the network. We can then compute temporal offsets by slightly expanding how we define the refractory period and signaling latency. For the refractory period, we let *ϕ*_*j*_ represent a temporal offset from *R*_*j*_, such that at *T*_*o*_.

2 $${\overline{R}}_{j}={R}_{j}-{\varphi }_{j}\,{\rm{where}}\,0\le {\varphi }_{j}\le {R}_{j}$$

We call ${\bar{R}}_{j}$ the effective refractory period. It reflects the amount of time remaining in the recovery from *R*_*j*_ at the observation time *T*_*o*_.

Similarly, the times at which each *v*_*i*_ vertex initiates a signaling event would not be expected to be all the same. At any given arbitrary observation time *T*_*o*_ a signal from any *v*_*i*_ may be traveling part way along *e*_*ij*_ at a speed *s*_*ij*_, effectively shortening *τ*_*ij*_. Or it may be delayed in signaling if *v*_*i*_ signals some time after *T*_*o*_, effectively lengthening *τ*_*ij*_. At *T*_*o*_ we need to take into account the degree of signaling progression for each *v*_*i*_ along its edge. To accomplish this, we extend how we consider a signaling latency in the following way. First, we retain *τ*_*ij*_ to represent the absolute temporal delay (latency period) for a signal that travels on the edge *e*_*ij*_ for a vertex *v*_*i*_. Note that the initiation of the signaling event could come before, right at, or after the observation time *T*_*o*_. We then considered a temporal offset for *τ*_*ij*_, an effective shortening or lengthening of *τ*_*ij*_ relative to *T*_*o*_ as follows

3 $${\bar{\tau }}_{ij}={\tau }_{ij}+{\delta }_{ij}\,{\rm{where}},\,{\delta }_{ij}\in {\mathbb{R}}$$

*δ*_*ij*_ > 0 represents an effective delay or elongation beyond *τ*_*ij*_. In other words, it represents the vertex *v*_*i*_ initiating a signal at some time after *T*_*o*_. Values −*τ*_*ij*_ < *δ*_*ij*_ < 0 represent an effective shortening of *τ*_*ij*_. This would be the case when *v*_*i*_ had initiated a signal that was traveling part way along the edge *e*_*ij*_ towards *v*_*j*_ prior to *T*_*o*_. When *δ*_*ij*_ = 0 it implies that *v*_*i*_ signals exactly at the moment the network is observed. And when *δ*_*ij*_ = −*τ*_*ij*_ it implies that the signal arrives at *v*_*j*_ at the moment the network is observed. Values of *δ*_*ij*_ < *τ*_*ij*_, which result in ${\bar{\tau }}_{ij} < 0$, represent a signal arriving at *v*_*j*_ *prior* to the observation time *T*_*o*_.

In the general case then, we were then able to extend Equation 1 to reflect the effective refractory period and effective latency (relative to an observation time *T*_*o*_) as

4 $${{\rm{\Lambda }}}_{ij}=\frac{{\bar{R}}_{j}}{{\bar{\tau }}_{ij}}$$

### Efficient signaling using the refraction ratio

We then defined a notion of optimized efficient signaling in the context of the competitive refractory dynamic framework and the refraction ratio. Given an effective refractory period ${\overline{R}}_{j}$ and effective latency ${\overline{\tau }}_{ij}$ along an edge *e*_*ij*_, the condition for the winning vertex *v*_*i*_ from the set of vertices that connect into *v*_*j*_ that achieves activation of *v*_*j*_ is dependent on the ${\mathrm{lim}}_{{\bar{\tau }}_{ij}\to {\bar{R}}_{j}^{+}}$ for ${{\rm{\Lambda }}}_{ij}={\overline{R}}_{j}/{\overline{\tau }}_{ij}$. In other words, the first discrete signal that arrives at *v*_*j*_ after it stops being refractory is the signal that results in its activation. Intuitively, efficient signaling in the context of the framework means that there should be a temporal match between the dynamics of signals traveling on edges relative to how quickly nodes can internally process signals. When a mismatch between these two considerations occurs, it can cause a break down in the signaling dynamics of the network. If signaling speeds *s*_*ij*_ are too fast, or equivalently, if the latencies ${\overline{\tau }}_{ij}$ are too short, compared to the refractory period of the node, the network will not be able to sustain recurrent activity^17^. If *s*_*ij*_ is too slow or the set of ${\overline{\tau }}_{ij}$ too long then the network will be inefficient in the sense that it has the potential for faster dynamic signaling that is not being realized. Time, as a resource, is being wasted in such a network. In^16^ we formalized these concepts by deriving upper and lower bounds for the refraction ratio between connected vertices *v*_*i*_ and *v*_*j*_. We were able to show that, given a subgraph of the network as a set of nodes *v*_*i*_ with directed edges into a node *v*_*j*_, the optimal refraction ratio [Λ_*ij*_]_*opt*_ is bounded by

5a $${[{{\rm{\Lambda }}}_{ij}]}_{opt}=\mathop{\mathrm{lim}}\limits_{{\tau }_{ij}\to {R}_{j}^{+}}{{\rm{\Lambda }}}_{ij}\,{\rm{when}}\,{\varphi }_{j}\,{\rm{and}}\,{\delta }_{ij}=0\,\,\,\,[\mathrm{Upperbound}]$$

5b $${[{{\rm{\Lambda }}}_{ij}]}_{opt}\Rightarrow \mathop{\mathrm{lim}}\limits_{{\delta }_{ij}\to -{\phi }_{j}^{+}}{{\rm{\Lambda }}}_{ij}\,{\rm{when}}\,{\varphi }_{j}={R}_{j}\,\,\,\,\,[\mathrm{Lowerbound}]$$

where *τ*_*ij*_ is the absolute signaling delay on the edge *e*_*ij*_ and *R*_*j*_ is the absolute refractory period for *v*_*j*_. Given these bounds, an optimized refraction ratio is such that

5c $${[{{\rm{\Lambda }}}_{ij}]}_{opt}=\frac{{\bar{R}}_{j}}{{\bar{\tau }}_{ij}}\to 1.$$

We refer the reader to^16^ for the full derivation and proof.

### Refraction ratio analysis in axon arbors

In this paper, we considered the morphological structure of individual Basket cell neurons as geometric networks, and carried out an analysis of the refraction ratio for individual axonal branches given experimentally determined morphologies and computed values of conduction velocities and refractory periods. Specifically, we considered a path consisting of the soma of a neuron and its axon and axonal arborizations. We first mapped the axon arbor network into a tree-graph consisting of a root vertex defined at the axon’s initial segment, and terminal vertices at each synaptic terminal (see Methods section). We then evaluated the signaling efficiency at synaptic terminals by computing the refraction ratio at each terminal vertex as a function of the geometry (morphology) of the respective axonal branches. We use the term ‘branch’ to refer to the sequence of axonal segments connecting the root vertex, i.e. the first vertex at the soma, to a specific terminal vertex at one of the axon's synaptic terminals.

The refraction ratio is a point property, in the sense that it is computed at a specific node in the network. We computed the ratio for a known set of defined nodes corresponding to specific axonal segments along the length of a branch, culminating at the terminal synaptic node. Computing the ratio depends on knowledge or estimation of the conduction velocity of the action potential and the refractory period for the node under consideration. The refractory period or refractory state of individual nodes along specific points in the arbor graph reflect the refractory period of the membrane at the corresponding points along the axon (see the Methods section for full details regarding the estimation of the refractory period). The refraction ratio along the defined node points of the branching path that connects the root vertex to the synaptic terminal vertex is a property of that branch. It depends on the time of propagation of an action potential along that branch (which is a function of the morphology and conduction velocity). Within this context, the refraction ratio determines a gating effect at each node: in setting the rate at which each action potential can be transmitted along an axonal branch, and as a result when synaptic signaling occurs, it dictates the frequency at which each neuron fires.

## Results

### Graph-Based Model of Geometric Axon Arbor Networks

**Figure 1:**
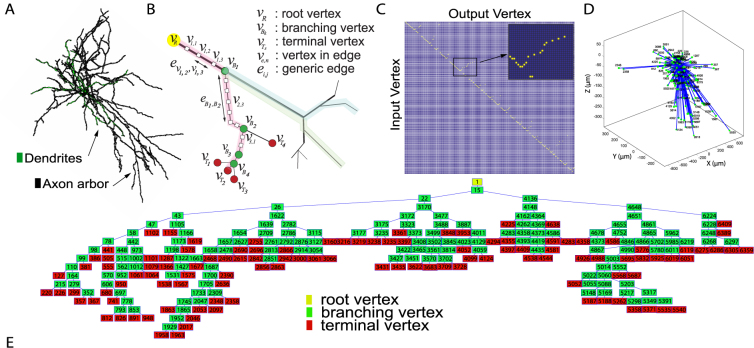
Deriving axonal network tree graphs from morphological reconstructions. (**A**) Representative three dimensional morphological reconstruction of one of the Basket cells in our data set from rat neocortex. Dendritic arbors are in green, axonal arbors in black [data from http://neuromorpho.org/^21–23^]. (**B**) Graph-based model of the geometric network of an axon and its arborizations. Vertices in the graph are labeled as follows: root vertex *v*_*R*_ is located at the initial segment of the axon at the soma; branching vertices *v*_*B*_ corresponding to branch points along the axons; terminal vertices *v*_*T*_ were synaptic terminals of the axon arborizations. Vertices between branch points reflect measurement points in the original reconstruction, and are denoted as *v*_*e*,*n*_, with *e* identifying the edge and *n* the specific axonal point within that edge. The computation of the convoluted geometric distance between the root vertex *v*_*R*_ and a selected terminal vertex ${v}_{{T}_{s}}$ was determined by the sum of all edge distances between *v*_*e*,*n*_ pairs forming the total path that connected the two vertices. The total distance approximates well the edge path length integral (see the Methods) of the tortuous path connecting the axon initial segment to a synaptic terminal in a real neuron (see panel A). (**C**) Reduced adjacency matrix mapping the connectivity between branching points along the axon tree of the neuron visualized in A. (**D**) Three dimensional graph of the same axon arbor mapped onto a minimized length network where only root vertex and terminal vertices are considered. The units are microns, as indicated. Metric distances are Euclidean distances from the root vertex at the center of the figure (common point for each branch) to the axonal terminals. The actual terminals are the green points in the figure. The 3D positions of root and terminal vertices (X, Y, Z coordinate values) were obtained from the 3D morphological reconstruction in the NeuroMorpho database. Visually, Euclidean distances are represented by the blue lines connecting the root to the terminal vertices. (**E**) Biograph of the reduced network of the axon arbor in A, described by the adjacency matrix in C. Each labeled box in the graph stands for an identification number of the reported vertex. Root vertex in yellow, branching vertices in green, and terminal vertices in red.

We carried out our analysis of the refraction ratio in geometric tree networks of morphologically reconstructed axonal arbors. In order to compare our results with those of Budd and colleagues^15^, we choose to analyze Basket cell neurons. Specifically, we considered anatomical and morphological data from detailed three-dimensional (3D) reconstructions of complete single neuron axonal arbors of 57 basket cells from the rat neocortex^21–23^. This resulted in a data set consisting of 11,575 independent axonal branches. The morphology of one of the basket cells we analyzed is shown in Fig. 1A.

We used a graph-based modeling approach to map the geometric connectivity of the axon arbor structures. The vertices in the graph reflect sampling points in the original morphological reconstruction of the neurons, and are associated with a physical position in three dimensional space (see NeuroMorpho.Org^22,23^). Figure 1B shows a graphical representation of the axon tree network: the root vertex *v*_*R*_ corresponds to the initial vertex at the axon initial segment where the axon begins at the base of the cell body (soma); branching points along the axons were labeled as branching or bifurcation vertices *v*_*B*_; synaptic terminals were defined as fixed end terminal vertices *v*_*T*_. We use the term ‘branch’ to refer to the axonal segment connecting the root vertex, i.e. the first vertex at the soma, to a specific terminal vertex at one of the axon’s synaptic terminals. Vertices in between these two end point vertices reflect axonal segments along the way, defined by the original morphological sampling frequency of the data in the NeuroMorpho database. We call these elementary vertices *v*_*e*,*n*_. We equivalently use the term ‘branch’ or ‘branching path’ in some instances in the paper. Within a specific neuron, every branch shares a common subset of vertices, specifically, the vertices for the segments that make up the axon itself from the soma up to where the axonal arborization branches off into individual synaptic branches. A full branching path then, is composed of this common axonal subset plus the unique arborization branch that ends at its respective synaptic terminal. Mathematically, we summarize a specific branch as $p({v}_{R},\,{v}_{{T}_{s}})$. For example, in Fig. 1B, a branch is the sequence of axonal segments that connects *v*_*R*_ to the terminal vertex ${v}_{{T}_{1}}$. A second branch is represented by the sequence of axonal segments that connects *v*_*R*_ to the terminal vertex ${v}_{{T}_{2}}$. In other words, a branching path $p({v}_{R},\,{v}_{{T}_{s}})$ is the path through which an action potential that is generated at the soma propagates to reach a specific synaptic terminal.

Figure 1C shows the adjacency matrix encoding all the edges for the network graph representation of the neuron. A pair of vertices (*v*_*i*_, *v*_*j*_) in the graph are connected by a directed geometric edge *e*_*ij*_ that reflects the morphological geometry of the axonal segment linking both vertices. Each edge is associated with a length parameter associated with the convoluted length of the axonal segment in the arbor, computed as the path integral of the corresponding curve. Additional details about network construction, as well as the description of the edge path length integral calculation can be found in the Methods.

Figure 1D plots the three dimensional graph of the axon network. For clarity, this particular plot only shows the root vertex *v*_*R*_ and synaptic terminals *v*_*T*_. The plot shows the length minimized network, where the distance from root vertex to terminals is the shortest straight line path calculated as the Euclidean distance. The figure highlights the difference between the geodesic (shortest) path distance in the minimized network, relative to the actual morphological paths of the axon arbor (Fig. 1A). Panel E in Fig. 1 shows the biograph of the same axonal network. We observed similar single neuron reconstructions for all the cells in our data set.

### Signaling Dynamics in Axon Arbor Networks

We characterized the signaling dynamics on individual axonal arbors by estimating the conduction velocities (signaling speeds) of action potentials, and computing the propagation delays (signaling latencies) for each branch. The axonal branches were defined by fixing the principal vertex *v*_*R*_ and each terminal vertex ${v}_{{T}_{s}}$ in the axon graph for all branching paths $p({v}_{R},\,{v}_{{T}_{s}})$ (the subscript *s* indexes specific vertices - see Methods). Axon conduction velocity is a function of the biophysical and morphological properties of the membrane and axon; it varies with axon thickness, branching, ion channel density and variety, and myelination^24^. We assumed that the neurons we analyzed here were unmyelinated, inferred from their small average diameter (mean 0.63 *μ*m) from direct measurements^25^ and theoretical studies^26,27^ (see the Methods for a detailed justification and calculations). We then derived the conduction velocity *s*_*ij*_ between any two vertices *v*_*i*_ and *v*_*j*_ as a function of the axonal diameter *D* according to the relationship in^28^. We estimated an average conduction velocity for each individual axonal branch separately as described in detail in the Methods.

**Figure 2:**
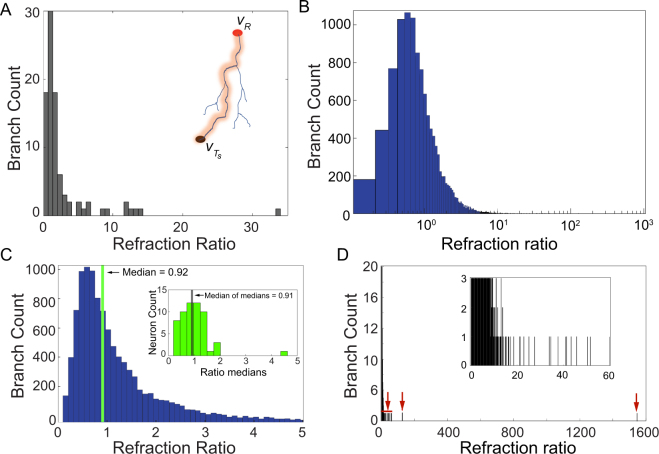
Basket cell neurons display optimized refraction ratios. (**A**) Distribution of the refraction ratio for all branches forming the axon arbor for a single neuron, i.e., for all paths *p*(*v*_*R*_, *v*_*T*_) connecting the root vertex at the initial segment *v*_*R*_ to the synaptic terminals *v*_*T*_. The inset shows an example of one axonal branch path from this cell. The refraction ratio was computed independently for each axonal branch for all branches of the axon tree. The *x*-axes represents values of the computed refraction ratio, while the *y*-axes shows the number or count of individual axonal branches. (**B**) Distribution of the refraction ratio for the full data set of 57 basket cells. The refraction ratio axis is on a logarithmic scale. All axonal branches (11,575) from all the neurons are statistically independent variables, and the value of the refraction ratio corresponding to each of them was used to build the distribution. (**C**) The peak of the distribution shown in B occurred at 0.56, while the median had a value of 0.92. The median value of all medians computed for each of the 57 neurons in the data set had a value of 0.91 (see the discussion in the main text). Inset: histogram of the median values of the refraction ratio distribution computed for each of the 57 neurons. The computed mean of the median ratio distribution had a value of 1.01 with 49 neurons (86 % of the entire data set) within one standard deviation, 1.01 ± 0.61 (*μ* ± 1*σ*). (**D**) Distribution of the refraction ratio for the entire data set (same data shown in panel B) but with *x*-axis extending out to the full range of values in order to show the few outliers (indicated by the red arrows). Inset: zoomed in view of the main plot out to a refraction ratio of 60 which captures the range over which most of the outliers were found. Note how the branch count steeply decreases from the peak. The distribution of refraction ratio values, the number and width of each bin, was obtained by dividing the full range of the computed refraction ratio by the smallest value of the ratio in order to obtain reasonable bin sizes (see Methods).

We obtained an average conduction velocity of 0.39 ± 0.16 m/s (Fig. 2 in Methods). Current estimates of mean intracortical axonal conduction velocity in adult cat visual cortex vary in the range 0.1–0.6 m/s^29–31^ with similar values reported for horizontal axonal connections in rat neocortex (0.3–0.5 m/s)^25,32,33^. It is important to note that the accuracy of axonal diameter values are dependent on the resolution of the imaging techniques used in^21^. Fractions of microns are not easily or reliably measurable by optical microscopy. This uncertainty on the variability of the diameter data was accounted for in our analysis by computing average conduction velocities.

To estimate the signaling latency *τ*_*ij*_ on an edge *e*_*ij*_, we divided the path length |*e*_*ij*_| between vertices *v*_*i*_ to *v*_*j*_ by a conduction velocity *s*_*ij*_. The labels *v*_*i*_ and *v*_*j*_ indicate any vertex pair with *v*_*i*_ connecting into *v*_*j*_ regardless of the type of vertex (i.e. *v*_*R*_, *v*_*T*_ or *v*_*e*,*n*_). We then computed the total propagation delay along an axonal branching path $p({v}_{R},\,{v}_{{T}_{s}})$ as the sum of all temporal delays for each individually computed segment (see Methods).

### Refractory Dynamics in Basket Cell Axon Arborizations

We next independently computed the refractory period *R*_*j*_ at the positions on the membrane corresponding to vertices *v*_*j*_ for every axonal segment in the tree network of all 57 neurons. Accurate experimental measurements of sub-type specific axonal membrane refractory periods are difficult to find in the literature. In general, refractory periods are defined as a range of values for whole neurons and almost always without regard for differences along different axonal segments. Textbook values of the absolute refractory period are typically reported to range between 0.8–1 ms^34^. The relative refractory period typically lasts up to 5 ms. A few studies have investigated the details of the refractory period in the context of specific morphological and biophysical measurements^35–38^. This has allowed other investigators to study the role of axonal refractoriness on the signaling dynamics and information processing along individual axon branches, in response to extracellular activation^39,40^. However, although it is known that membrane refractoriness can change along the length of the axon and its axonal branches, there is limited empirical data available^41^. Recent work by Hu and colleagues on the biophysical and dynamical properties of Basket cells, the same class of neurons we investigated here, allowed us to estimate changes in the refractory period along axon arbors^25^. These authors showed that Basket cells display a supercritical density of sodium ion (Na^+^) channels with a specific density profile along the axonal tree, responsible for high reliability fast propagation in nonmyelinated small axons. Additionally, analysis of Na^+^ channel gating kinetics and voltage-dependence of activation and inactivation revealed that the inactivation time constant was 2.4-fold faster for axonal versus somatic Na^+^ channels. Based on these observations, we estimated individual values of the refractory period *R*_*j*_ at axonal branching points as a function of the distance from the soma. We computed variations in the membrane refractory period using a derived function inferred from actual measurements of channel density variations in these neurons^25^ (see Supporting Information).

### Basket Cells Axon Branches Approach Near Optimal Values of the Refraction Ratio

The refraction ratio is defined as the numerical ratio between the refractory state *R*_*j*_ of a vertex *v*_*j*_, and the temporal propagation delay (signaling latency) *τ*_*ij*_ of a signal traveling from a vertex *v*_*i*_ that connects into *v*_*j*_^16^. We calculated the refraction ratio for each of the 11,575 axonal branch paths *p*(*v*_*R*_, *v*_*T*_) from root vertex *v*_*R*_ to terminal vertices *v*_*T*_ independently for the entire data set. To compute the ratio, we used the path length, and calculated *τ*_*ij*_ and *R*_*j*_ values. Figure 2A, shows the distribution of the ratio for all branches of the axon arbor of one representative Basket cell from our data set. There is a clear clustering of the ratio near unity for the axonal branches of this neuron. The histogram for the distribution of refraction ratios across the entire data set for all the axonal branches is shown in Fig. 2B. There is a narrow peak in the distribution centered near unity. Figure 2C shows a highly magnified view highlighting the peak of the histogram in panel B. Approximately 76% (8, 795) of the total 11,575 branches had computed refraction ratios within the range of 0.25–1.75, with a median value of 0.92 for the entire distribution.

The value of the refraction ratio for the median of the medians across all 57 neurons had a computed value of 0.91 (inset in Fig. 2C). To compute the median of the medians we took the median value of the refraction ratio for each of the 57 basket cells computed independently from their respective distributions as shown for the representative neuron in panel A, and then obtained the median value of all the individual medians across the entire population. Our rationale for computing the median of the medians was that it provides a normalization against the varying total number of terminal vertices (synapses) across our sample population of 57 neurons. The median of the entire distribution pools all the ratios equally for all computed axonal branches across the whole population, but it does not take into account that different neurons contribute different numbers of ratios to the distribution because they have more (or less) number of axonal branches. This means that neurons that have more branches will have a greater effect on the value of the median of the population, and if they happen to have a disproportionately greater number of outlier ratio values these outliers will skew the value of the median. By computing the median of each cell independently, and then identifying the median of the population of median values, each of the 57 neurons have equal weight or effect on the median of the medians, regardless of the total number of terminal vertices they may have. It effectively filters out or normalizes a disproportionate effect contributed by outlier ratio values. Interestingly, the correspondence of the median of the entire population versus the median of the medians was almost identical (0.92 versus 0.91), adding further weight to the validity of the results.

In addition to the median values being near the theoretically predicted optimal, the distribution of the population of the medians, the degree of clustering near the peak, is equally significant. Because each median was computed independently for each neuron, the population of median values can be treated as its own data set. Of the 57 neurons, 49 of them (86 %) had ratios falling within one standard deviation, within the range 1.01 ± 0.61 (*μ* ± 1*σ*). Nearly the entire sampled population, 56 out of 57 neurons, had median values of their ratios falling within two standard deviations, in the range 1.01 ± 1.22 (*μ* ± 2*σ*) for the positive values of the ratio (since negative values have no meaning).

Importantly however, we note that some axonal branches did deviate significantly from an optimized refraction ratio, in one case extending out to an extreme value of 1600; see the outliers in Fig. 2B and D. This lends support for the sensitivity of the refraction ratio as a metric of signaling efficiency in neurons. We discuss the possible functional implications of such outliers in the Discussion below.

### Morphological Regulation of Signaling Latencies

**Figure 3:**
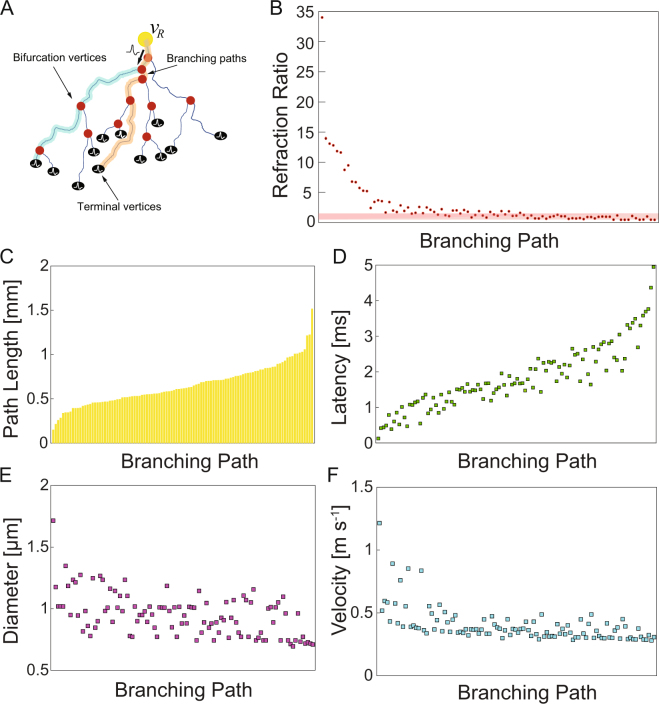
Effect of axonal morphological branch path lengths and conduction velocities on computed refraction ratios. (**A**) Stylized axon arbor of a neuron. Yellow circle: root vertex at the axon initial segment; red circles: bifurcation vertices; black circles: terminal vertices at the axon’s synaptic terminals. A branching path $p({v}_{R},\,{v}_{{T}_{s}})$ is defined as the axon segment connecting the root vertex *v*_*R*_ to a specific terminal vertex ${v}_{{T}_{s}}$. The two example branching paths in the figure (blue and orange lines) share the first two bifurcation points and corresponding edges. An action potential is generated at the axon initial segment and propagates from the soma to each synaptic vertices throughout the individual branching paths of the arbor. (**B**) Refraction ratios computed for all branches of the axonal tree of one representative basket cell. Each circle represents one individual path $p({v}_{R},{v}_{{T}_{s}})$. Data points within the red band are ratios within a range of 0.5 to 1.5, indicating branches that fell near an optimal value of unity. (**C**) Path lengths associated with the axon morphologies responsible for the convoluted edges connecting the root vertex at the initial segment (*v*_*R*_) to the synaptic terminals (*v*_*T*_). Each bar represents a different synaptic terminal (branch) of the axon tree. (**D**) Propagation delays (latencies) for the same cell. (**E**) Mean axonal diameter for each branch. The mean diameter for a given branch was calculated as the mean for all measured diameter points for each branch. (**F**) Signaling speeds (conduction velocities) for each branch in this neuron. Although branch path lengths varied significantly, conduction velocities for most branches were within a range of 0.3–0.5 m/s. The data for panels A-D on the *x*-axes were ordered by path length from shortest to longest.

In order to investigate how the different parameters that define the refraction ratio affected the computed values for our data, we looked more closely at what parameters were having the greatest effect on the axonal branches of one representative neuron. Figure 3A shows a stylized axon arbor and schematically exemplifies observed signaling pathways in our neuron graph model. An action potential is generated at the axon initial segment (*v*_*R*_, yellow circle) and propagates to individual synaptic vertices (terminal vertices *v*_*T*_, black circles) along the respective branching paths $p({v}_{R},\,{v}_{{T}_{s}})$ of the arbor. Two example branching paths are illustrated in the figure (blue and orange lines), which share the first two bifurcation points (red circles) and corresponding edges. Figure 3B shows the refraction ratios for all the branching paths $p({v}_{R},\,{v}_{{T}_{s}})$ of an individual cell ordered by decreasing edge path length (Methods). Similar to the results above, the plot shows how a large subset of the terminal vertices in this neuron fall within a range of 1 ± 0.5 (red horizontal bar in Fig. 3B). The value of the ratio is sensitive to and inversely related to the path length from the root vertex at the soma to the terminal vertices (Fig. 3C). Figure 3D shows the signaling latencies from the root vertex to the terminal vertices for all paths in the arbor. Signaling latencies are sensitive to |*e*_*ij*_|, which are determined by the axon’s morphology. These results are in agreement with numerical simulations of intracortical axon arbors which suggest that the path length is the main determinant of signaling latency^15^. Axonal branches longer than ~500 *μm* in this neuron were almost always capable of achieving an optimal ratio despite significant differences in morphological path lengths and signal latencies. We observed similar results in other individual neurons we looked at.

Figure 3E shows the relationship between the mean diameter measured as a function of position along individual branches of the axonal arbor, while Fig. 3F shows the scatter plot of the conduction velocity from the soma to its synaptic terminal along individual axonal branches. In contrast to the effect of the morphology (path length) there is comparatively little variability in the diameter, even though it is a key contributor to the conduction velocity. This is reflected in the computed constant conduction velocity across all branches of the neuron. We also showed that the convergence towards an optimal ratio occurs quickly, within a few branching orders from the soma (see the Supplementary Information).

### Perturbations of Morphological Path Lengths Resulted in Deviations of Optimal Ratios

**Figure 4:**
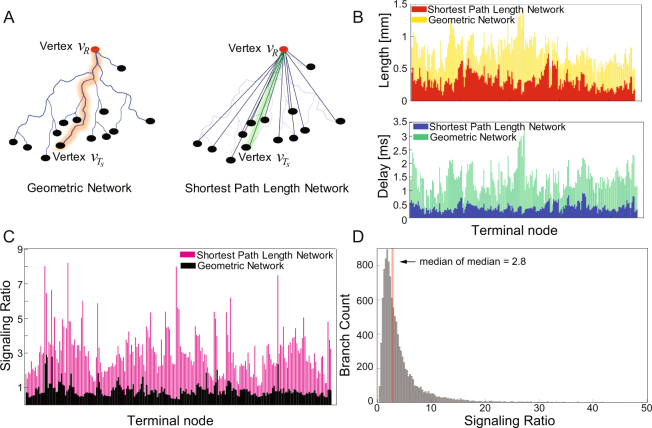
Effect of axonal branch path length on the refraction ratio. (**A**) As a control, we re-computed the refraction ratio for the entire data set with axonal branch path lengths that reflected the shortest Euclidean distance between the root vertex and terminal synaptic vertices. Left: The geometric tree network for one representative neuron where each edge connecting the root vertex to the terminal synaptic vertices were associated with the geometric convoluted path length (see the Methods). Right: the path length minimized tree network for the same neuron, where the length of the edges connecting the vertices were defined by the shortest Euclidean distance. (**B**) (Top) Path length computation in the geometric network (yellow bars) superimposed on the path length minimized network (red bars) for one of the 57 axon arbors. Each bar in the diagram identifies a different axonal branch. (Bottom) Signaling latencies derived for the geometric network (green bars) and the minimized network (blue bars) for the same axon tree. (**C**) Computed refraction ratios for the geometric network versus the minimized network for the same cell as in (**B** and **C**). The height of each of the black bars stands for the refraction ratio calculated as a function of the convoluted geometric path of an individual branch of the axon. (**D**) Histogram showing the global distribution of refraction ratios computed for all 11,575 branches of 57 neurons in our data set when mapped into a length-minimized network. The median value of all the medians computed for length minimized networks for all the neurons was centered at 2.8, indicating a deviation from optimality of the refraction ratio as a result of the perturbed path lengths associated with ignoring the geometry of the axonal morphologies.

We then investigated the effect that changing the axonal path lengths has on the ability of the neuron to compensate for longer latencies in order to preserve a near optimized refraction ratio at synaptic terminals. We compared ratios for the actual geometric morphology of a representative neuron (Fig. 4A, left) to a length-minimized version of the same cell (Fig. 4A, right) constructed by measuring the Euclidean (shortest) distance between the root vertex at the soma and terminal vertices at the synaptic terminals (cf. Fig. 1D). Figure 4B shows the distribution of lengths (distance of terminal vertex from the root vertex) for all branches in the tree network for this neuron and the distribution of signaling latencies, respectively. Figure 4C shows the refraction ratios for all paths between the soma and synaptic terminals of the same Basket cell calculated for the two different configurations of the network. As would be expected, for the length minimized network all branches have signaling ratios that deviate significantly from optimality. Figure 4D shows the refraction ratio for the entire 57 neurons and all individual axonal branches computed for length minimized tree networks. The population median of the medians of the ratios increased from 0.92 to 2.8. Mechanistically, this is due to poor temporal matching between signaling latencies and refractory recovery times at the synaptic terminals. The path length minimized networks still preserve the relative order of the axonal branches organized by lengths, in the sense that longer branches relative to shorter branches in the morphologically accurate geometric networks will still be longer in the shortest path networks. But the entire distribution of the ratios is skewed.

## Discussion

In this work we investigated the relationship between axonal morphology, membrane refractory period, and action potential conduction velocities through an analysis of computed refraction ratios. This analysis allowed us to understand how the interplay between structure and dynamics achieves signaling efficiency in Basket cell neurons. Presumably, either directly or as a consequence of other processes, neurons are optimizing the refraction ratio as a design target.

Of the total 11,575 axon branches we computed refraction ratios for, 76% had a ratio within a range 0.25–1.75. 86% of the neurons had median ratio values within one standard deviation of the median of the population (0.91); a value that approaches the theoretically predicted maximal efficiency. This suggests that while an optimal refraction ratio at synaptic terminals is a local property associated with individual axonal arborizations, it is highly conserved across the entire population of sampled neurons.

In some cases, we also showed that some axonal branches did deviate significantly from an optimized refraction ratio (Fig. 2D). Some amount of variability is to be expected from any analysis of empirically measured experimental data. However, we note that the number of outliers was very small. An interesting question is whether these reflect ‘noise’ in the data set, essentially true (random) outlier events, or if they instead perhaps exhibit such extreme refraction ratios for a physiological reason. For example, one possibility is that these outliers reflect axonal branches that are designed to filter specific signals at a subset of synaptic terminals. The presence of such outliers also serve the purpose of validating the sensitivity of the refraction ratio in capturing a mismatch between refractory states and signaling latencies within physiologically plausible bounds.

The analysis also suggests that morphological regulation of signaling latencies exerts the greatest control on the refraction ratio. The data in Fig. 3 indicates that the shortest branches are not able to preserve an optimal ratio. This is because the latencies are too short and not able to match the refractory period at such short distances from the soma. One interpretation is that these branches are outliers relative to the rest of the dataset. This is statistically reasonable if we consider the variability of the experimental parameters. Incomplete development of axon arbors or incomplete pruning associated with the young age of the rat from which our data were derived (10–15 days postnatal) could possibly account for the discrepancies in the refraction ratio values. However, axons can also make synaptic contacts at short distances for physiologically functional purposes. Taking this into consideration, these outliers could be possibly serving a physiological role. One possibility is that branches that display non-optimality of the refraction ratio could be playing a different function in the dynamics of the cell, such as the propagation and transmission of signals at lower or higher frequencies. Such a possibility for a functional role of non-optimal ratios is further supported by the fact that seemingly outlier refraction ratios showed similar trends in several neurons. In contrast to the effect of the morphology, there was less variability in the average diameter computed for different branches, even though it is a key contributor to conduction velocity. This is reflected in the computed constant conduction velocities across all branches of the neuron. Conduction velocities and membrane refractory periods are the direct result of membrane biophysics; more specifically, the permeability and channel kinetics of sodium and potassium ion channel populations and the inactivation kinetics of sodium channels, respectively. These are biophysical properties that a neuron does not have control over in a sense. They represent generic cellular and molecular properties that the neuron cannot change. An intriguing interpretation of our results is that neurons may take advantage of the morphological geometry of individual axonal branches in order to manipulate signaling latencies to match the refractory periods at the terminal vertices (synapses). This would represent a mechanism or ‘design criteria’ to optimize signaling transmission in the cell.

We also tested the hypothesis that Basket cell axon arbors are not minimized for either wiring length or conduction velocity latencies^15^. By wire minimizing the axonal morphology, we were able to show that axonal path length is used to compensate for longer latencies in order to preserve a near optimized refraction ratio at synaptic terminals (Fig. 4). More significant deviations in the refraction ratios will occur when additional effects to axon path lengths and conduction velocities are considered, such as a reduction of the arbor diameter or randomly generated arbor graphs, as some authors have modeled^13,42–44^. In fact, shortest distance wiring path lengths like we computed here reflect the most conservative changes possible, and are therefore the least disruptive to the computation of the refraction ratios. This makes the surprising precision of the refraction ratio results near optimality for the morphologically accurate data even more significant.

Subsequent work should explore if an optimized refraction ratio holds for other types of neurons. We are also beginning to investigate experimentally if this principle holds in individual neurons where morphological reconstructions and detailed empirically measured values of conduction velocities and refractory periods across different segments are obtained in the same cell. Recent advancements in the development of novel electrophysiology techniques able to provide signal detection with high spatio-temporal resolution^45,46^ will greatly benifit this type of analysis by reducing the number of assumptions, such as computing rather than measuring refractory periods and conduction velocities that contribute to signaling latencies. Finally, beyond establishing the refraction ratio as the design principle being optimized by neurons are questions about the physiological purpose of preserving an optimized ratio. Presumably neurons are exerting this level of dynamic precision for important neurophysiological reasons that require further investigation. One example could be temporal precision at synaptic terminals and its consequences on signaling integration. This has been previously suggested as one reason why neurons are neither optimized for wiring length or signaling speeds^15^.

## Methods

### Axon Database

We used anatomical and morphological data from detailed three-dimensional (3D) reconstructions of complete axonal arbors of Basket cells from the rat neocortex^21^. Data were obtained from the online digital database NeuroMorpho.Org^23^. The neurons in our dataset were chosen based on the availability of high accuracy measurements of axon thickness or diameter throughout the whole arbor structure. More specifically, the data in the NeuroMorph database is comprised of spatial reconstructions and measurements along the axonal arbor, with an identification number for each sample point, a three dimensional location (*x*_*i*_, *y*_*i*_, *z*_*i*_), and the diameter *D*_*i*_ of the axon at that point. The database also contains indices indicating the physical connectivity between sample points along the arbors, thus providing all the relationships to construct the axonal tree. Different neurons have differing degrees with respect to the completeness of this information. Because of our analysis necessitated high detailed reconstructions we randomly selected Basket cell neurons that had the most complete information available.

### Axon Graph Description

#### Graph-model of axon arbors

**Figure 5:**
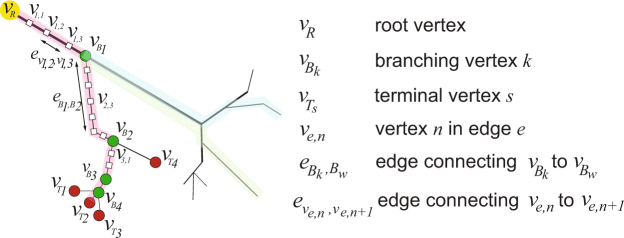
Graph-based model of the geometric network of an axon arbor.

We used a graph theoretic approach to describe the three dimensional connectivity of single axons. A schematic description of the graph model is shown in Fig. 5. An axon arbor is naturally modeled as a tree-graph. We used the term axon ‘branch’ or ‘branching path’ to refer to the axonal segment connecting the axon initial segment to one of the axon’s synaptic terminals. In a graph-based model, a ‘branch’ is composed of multiple axonal segments stringed together by a set of edge connected vertices. For the purposes of our analysis, we needed to distinguish between three classes of vertices and their corresponding edges. For each individual neuron in our dataset, vertices and edges were defined based on specific axonal landmarks. These were the initial axonal segment, axonal branching points or bifurcations, and synaptic terminals. These features mapped to a root vertex *v*_*R*_, branching or bifurcation vertices ${v}_{{B}_{k}}$ (*k* = 1, …, *Q* − 1 with *Q* − 1 total number of branching vertices in the selected branch), and terminal vertices ${v}_{{T}_{s}}$ (*s* = 1, …, *W*, with *W* total number of synaptic terminals, or branches, in a given neuron), respectively. The vertex pair consisting of the root vertex *v*_*R*_ and a synaptic terminal vertex ${v}_{{T}_{s}}$ connected by the edge ${e}_{R,{V}_{{T}_{s}}}$ defined a branching path. We equivalently used the label $p({v}_{R},\,{v}_{{T}_{s}})$ to indicate a specific branching path. Successive bifurcation vertices, *i.e* one branching point and the next branching point in an axonal arborization, are given by ${v}_{{B}_{k}(i)}$ and ${v}_{{B}_{k(i+\mathrm{1)}}}$, along with their edges ${e}_{{B}_{k}i,{B}_{k}i+1}$, the segment of the axon that connects two bifurcation points. Vertices in between the root and terminal vertex reflect axonal segments along the way, defined by the original morphological sampling frequency of the data in the NeuroMorpho database. We called these elementary vertices. Elementary vertices correspond to neither a branching vertex, a terminal vertex, nor the root vertex. The elementary vertices located in between bifurcation vertices of the same axonal branch are labeled *v*_*e*,*n*_ where *e* = 1, ..., *Q* (*Q* is the total number of edges in a branch), and *n* = 1, ..., *N* − 1 (*N* − 1 is the total number of nodes along the *e*^*th*^ edge). Edges that connect successive elementary vertices *v*_*e*,*n*(*i*)_ and *v*_*e*,*n*(*i*+1)_ were labeled ${e}_{{v}_{e,n(i)},{v}_{e,n(i+\mathrm{1)}}}$. Note that ${e}_{R,{V}_{{T}_{s}}}$ and ${e}_{{B}_{k(i)},{B}_{k(i+\mathrm{1)}}}$ are composed from subsets of elementary edges ${e}_{{v}_{e,n(i)},{v}_{e,n(i+\mathrm{1)}}}$.

#### Adjacency matrix

From the structural data we built an adjacency matrix that encoded information about the connectivity of the network. The adjacency matrix was organized such that each element represents a connection (graph edge) between an input vertex (row) and an output vertex (column). The axon and axon arborizations are represented by tree-network graphs. The associated adjacency matrix describes the composite hierarchical organization of the tree, where a common axonal segment (‘parent segment’, identified by all elements on the diagonally-shaped line in the connectivity matrix) gives rise to a number of daughter axonal branchlets.

For the purpose of our analysis we chose to reduce the full adjacency matrix (containing nodes from the sets *v*_*R*_, *v*_*B*_, *v*_*T*_, and *v*_*e*,*n*_) to an adjacency matrix containing vertices from the sets *v*_*R*_, *v*_*B*_, and *v*_*T*_. It is this reduced matrix that we used in our analysis. This reduced adjacency matrix was created for three reasons. First, branching points define the variable paths of action potentials (signals) transmission through the axon. Second, in the more general case of myelinated axons, bifurcation points typically corresponds to unmyeliated locations where a node of Ranvier is present and thus where the signal propagates down the axon by saltatory conduction. This agrees with the methodology of our analysis which focuses on pairs of defined vertices that have an active role in neural signaling. Finally, synaptic terminals define the different paths of signaling in the axonal arbor and are thus necessary for comparisons between selected branches in the tree. And synaptic terminals represent active areas of the axon where synaptic transmission to one or multiple post-synaptic neurons occurs.

### Deriving the Path Lengths of Axons and Axonal Arborization Branches

As described, pairs of vertices (*v*_*i*_, *v*_*j*_) in the graph were connected by a directed geometric edge *e*_*i*,*j*_. Each vertex in the graph was associated with a physical position in three dimensional space, and each edge followed a convoluted geometric path in space determined by the actual physical morphology. Given an edge *e*_*i*,*j*_, the convoluted distance, i.e. the physical distance ${\tilde{d}}_{i,j}$ of the edge between any two vertices *v*_*i*_ and *v*_*j*_ can be defined by the path integral ${\tilde{d}}_{i,j}$ from *v*_*i*_ to *v*_*j*_. To compute it, we took into account all segments forming a particular edge, and then calculated the sum of all Euclidean distances between pairs of nodes for a given segment. For example, to calculate the convoluted distance (path integral) ${\tilde{d}}_{{B}_{k},{B}_{w}}$ from branching vertex ${v}_{{B}_{k}}$ to branching vertex ${v}_{{B}_{w}}$, we approximated the length by:

6 $${\tilde{d}}_{{B}_{k},{B}_{w}}=\sum _{N}{\bar{d}}_{{n}_{m},{n}_{q}}$$

where ${\bar{d}}_{{n}_{m},{n}_{q}}$ is the Euclidean distance $({\bar{d}}_{{n}_{m},{n}_{q}}=\sqrt{{({x}_{{n}_{m}}-{x}_{{n}_{q}})}^{2}+{({y}_{{n}_{m}}-{y}_{{n}_{q}})}^{2}+{({z}_{{n}_{m}}-{z}_{{n}_{q}})}^{2}})$ computed between pairs of nodes (${v}_{e,{n}_{m}},\,{v}_{e,{n}_{q}}$), and *N* is the number of sub-edges ${e}_{{n}_{m},{n}_{q}}$ forming ${e}_{{B}_{k},{B}_{w}}$.

The tortuous path connecting the root vertex *v*_*R*_ to a selected synaptic terminal ${v}_{{T}_{s}}$ was the sum of all convoluted distances ${\tilde{d}}_{{B}_{k},{B}_{w}}$:

7 $${\tilde{d}}_{R,{T}_{s}}=\sum _{Q}{\tilde{d}}_{{B}_{k},{B}_{w}}$$

where *Q* indicates the total number of edges ${e}_{{B}_{k},{B}_{w}}$ that compose the selected path $p({v}_{R},\,{v}_{{T}_{s}})$ in the axonal tree. For example, for the neuron represented by the graph in Fig. 5 (path highlighted in red), the total distance between the root vertex and the synaptic vertex ${v}_{{T}_{3}}$ was computed by summing up all distances of the edges ${e}_{{B}_{k},{B}_{w}}$ between the branching vertices along that path, which in turn were obtained by the sum of all distances of the multiple sub-edges ${e}_{{n}_{m},{n}_{q}}$ they were composed of.

### Calculations of Signaling Parameters

Axon conduction velocity is a function of several biophysical and morphological properties, including axon thickness, branching, ion channel density and variety, and myelination^24^. Considering the predominant role that axon thickness and degree of myelination have in defining the conduction velocity of action potential in mammalian axons^47^, in this work we derived estimated values of conduction velocity based on only these two structural factors. For our data, we assumed that the arbors of all our basket cell neurons were unmyelinated based on morphological empirical results and geometrically-based models from the literature^25–27^. While it is generally agreed that the conduction velocity of myelinated axons increases linearly with increasing diameter^37,48,49^, the situation for non-myelinated axons is less definitive. Theoretical arguments and empirical measurements on large invertebrate axons suggest that the conduction velocity of non-myelinated axons is proportional to the square root of the axonal cross-sectional diameter^27^. However, other results seem to suggest that such a square-root relationship at least in invertebrates oversimplifies the situation^50,51^. In the vertebrate nervous system however very fine non-myelinated axons were found to support a linear relationship^28^.

In our data, the diameter of the axons we studied were comparable to the fine non-myelinated axons in^28^. Therefore, we assumed a linear relationship between axonal diameter and conduction velocity (signaling speed) of non-myelinated axons. Hoffmeister *et al*. experimentally derived the linear relationship *s* = 0.24 * *C* between the circumference *C* of non-myelinated fibers and their signaling speed *s*. Our data had axonal segments expressed as diameters, not circumference. We therefore wrote the equivalent relationship: *s* = *KD* with *K* = 0.24 * *pi* = 0.75, where *s* is signaling speed, *D* is the axon diameter, *K* is a constant, and the relationship between circumference and diameter is *C* = 2 * *pi* * *D*. The same relationship has been used in other recent work to investigate the volume-latency trade-off in neocortical axons^52^.

We used this relationship to estimate the signaling speed *s*_*ij*_ between vertex pairs *v*_*i*_ and *v*_*j*_:

8 $${s}_{ij}=0.75D$$

where *D* is the (constant) diameter of the axonal segment connecting *v*_*i*_ and *v*_*j*_. If *v*_*i*_ and *v*_*j*_ are consecutive measurement sample vertices between branching points, *v*_*e*,*n*_, *D* was typically constant because *D*_*i*_ ~ *D*_*j*_ due to the sampling resolution of the original data. If *v*_*i*_ and *v*_*j*_ corresponded to two consecutive branching points, *D* was calculated as the arithmetic mean of the diameters of all sample points between successive vertices ${v}_{{B}_{k(i)}}$ and ${v}_{{B}_{k(i+\mathrm{1)}}}$. Considering that sampling points along individual edges of the axon were characterized by small diameter changes, the average represented a good approximation of the diameter of that particular axonal segment.

**Figure 6:**
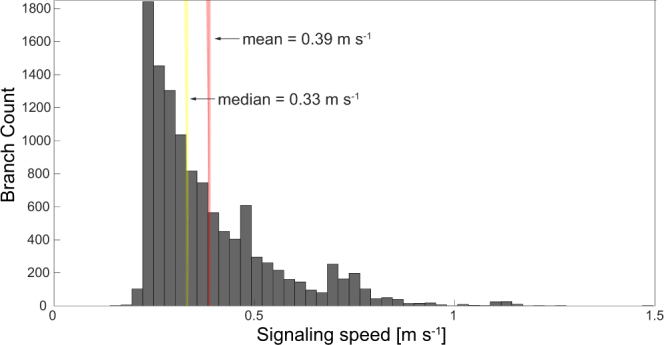
Global distribution of the signaling speeds independently computed for all branches of the 57 basket cells. Signaling speeds of individual axon branches range from 0.15 to 1.49 m/s (mean 0.39 m/s, median 0.33 m/s).

Neurons in our dataset had computed conduction velocity values of 0.39 ± 0.17 m/s (*μ* ± *σ*, with *μ* being the mean computed on the raw distribution of conduction velocities from all 11,575 axonal branches, and *σ* the standard deviation of their distribution). The histogram in Fig. 6 shows the distribution of signaling speeds independently computed for all branches of the entire data set of 57 basket cells. We also considered the distributions of conduction velocities for individual neurons in order to better characterize the behavior of single cells. For each neuron we computed the mean value of the conduction velocities of all axonal branches. We then calculated the mean value of the 57 computed means, obtaining a mean value of all means of 0.38 m/s.

The signaling latency or propagation delay *τ*_*ij*_ of action potentials (signals) traveling between vertices *v*_*i*_ and *v*_*j*_ were calculated as:

9 $${\tau }_{ij}=\frac{{d}_{ij}}{{s}_{ij}}$$

where *d*_*ij*_ is the distance measured between vertex *i* and vertex *j* as described in the previous section. Because our analysis is highly sensitive to the convoluted path length between vertices, the calculation of signaling speed and propagation delay depended on the kind of vertices that are considered in the arbor graph. For pairs of consecutive vertices *v*_*e*,*n*_ belonging to individual edges between branching vertices, the distance was calculated using the Euclidean distance between them, and the dynamic parameters, conduction velocity and signaling latency, were obtained according to Eqs 10 and 11. On the other hand, for vertices corresponding to branching points or synaptic terminals of the arbors, the dynamics was computed slightly differently taking into consideration that any of these convoluted edges can be decomposed into multiple sub-edges of uniform diameter and conduction velocity. More specifically, for each branch of the axon arbor, that is the path connecting the root vertex *v*_*R*_ to a synaptic terminal ${v}_{{T}_{s}}$, we calculated the total propagation delay as the sum of all piecewise propagation delays obtained for all edges ${e}_{{B}_{k},{B}_{w}}$ forming that branch:

10 $${\tau }_{R,{T}_{s}}=\sum _{Q}{\tau }_{{B}_{k},{B}_{w}}$$

with *Q* being the total number of edges connecting the initial segment to a synaptic terminal. In turn, each edge in the branch can be decomposed into multiple sub-edges ${e}_{{n}_{m},{n}_{q}}$ along which the signal propagates with a temporal latency defined as:

11 $${\tau }_{{B}_{k},{B}_{w}}=\sum _{N}{\tau }_{e,{n}_{m},{n}_{q}}$$

where *N* is the total number of sub-edges ${e}_{{n}_{m},{n}_{q}}$ in ${e}_{{B}_{k},{B}_{w}}$. We then used the information about axonal path lengths and latencies to compute the signaling speed. By doing this we avoided averaging different diameters in the chosen path for the calculation of a mean signaling speed. We instead considered multiple conduction velocities obtained for short segments between consecutive edges in the path that had a constant diameter *D*. We then used the resulting combination of propagation delays for estimating the signaling speed between the axon initial segment and its synaptic terminals. This more accurately reflects the true values of action potential propagation speeds.

### Estimation of the Refractory Period

The refractory period was estimated along the entire length of the axon, from the soma (root vertex) to each synaptic terminal (synaptic vertex). Since each path from root vertex to a terminal vertex was defined as a branching path, it is equivalent to saying that the refractory period was estimated for all axonal sampling points (i.e. nodes) along each specific branch, as a function of the distance between root and terminal vertex. We estimated the refractory period from empirical measurements reported in the literature^25^. More specifically, we approximated the decreasing refractory period from soma to synaptic terminals with a curve whose descending slope was inferred from actual measurements of channel density variation and membrane inactivation properties^25^. We set a lower bound to 1 ms, which is about the absolute lower limit for any neuron due to fundamental limitations associated with the kinetics of Na^+^ channels. The maximum value for *R*_*j*_ was set to 2.5 ms, which we justify by three specific arguments: first, 2.5 ms represents a reasonable value as the average of the generally accepted range (0–5 ms) associated with neocortical neurons^53–56^; second, work on demyelinated axons and the supercritical Na^+^ channel density proposed in^25^ implies that higher values would not be physically appropriate; finally, a change of refractory period from 1 ms to 2.5 ms is in agreement with the 2.4-fold increase of activation time for Na^+^ channels suggested by the experiments in^25^. A detailed explanation about the rationale for computation of a local refractory period for each vertex in the axon graph is reported in the online Supporting Information.

### Computation and Statistics of the Refraction Ratio

We considered the refraction ratio^16^ between all pairs vertex pairs (${v}_{R},\,{v}_{{T}_{s}}$): the root and terminal vertices in the network, the first vertex corresponding to the initial axon segment and the second to any synaptic terminal at the end of an axonal arborization. The value of the refraction ratio was obtained by computing the numerical ratio between the refractory period of any terminal vertex ${v}_{{T}_{s}}$ and the propagation delay signaling latency along the axonal branching path $p({v}_{R},\,{v}_{{T}_{s}})$ connecting the root vertex to the terminal vertex. The refraction ratio was computed independently for all reconstructed axon arbor graphs across each of the 57 basket cell neurons in our dataset. This resulted in 11,575 statistically independent refraction ratio values.

Histograms were used to collect, visualize, and analyze the data. At the single neuron level, each histogram described the distribution of the computed refraction ratio for all pairs of root and terminal vertices in the analyzed axon arbor (c.f. Fig. 2A). The value of bin within the distribution (the height of individual plotted bars) reflected the number of branches associated with refraction ratio value falling within a the specific range for the interval defined for that particular bin. For each basket cell we independently (from other neurons) computed the median value of the distribution.

To visualize and compute the statistics on the entire dataset, we constructed a histogram for the raw distribution of refraction ratio values computed for the 11,575 axonal branching paths from each of the 57 neurons (c.f. Fig. 2B). To normalize against varying numbers of terminal vertices across our sample population of 57 neurons, we computed the median value of the median population (see the main text above) (c.f. Fig. 2C).

## Electronic supplementary material

Supplementary Information
