# Supplementary material for: An Optimized Structure-Function Design Principle Underlies Efficient Signaling Dynamics in Neurons
Source: Sci Rep. 2018 Jul 11;8:10460. doi: 10.1038/s41598-018-28527-2 (PMC6041316; doi:10.1038/s41598-018-28527-2)
Supplement: Supplementary file 1 — Supplementary Information [file 41598_2018_28527_MOESM1_ESM.pdf]

Supporting Information

An Optimized Structure-Function Design Principle Underlies Efficient Signaling Dynamics in Neurons

Francesca Puppo[1,3], **Vivek George**[1,3], **Gabriel A. Silva**[1,2,3,*]

[1] Department of Bioengineering, University of California, San Diego, La Jolla, 92093 CA
[2] Department of Neurosciences, University of California, San Diego, La Jolla, 92093 CA
[3] Center for Engineered Natural Intelligence, University of California, San Diego, La Jolla, 92093 CA
[*] Email: gsilva@ucsd.edu

Estimation of the Refractory Period

Our rationale for justifying computing a local refractory period for each vertex was based on the following considerations. The inactive state of a neuron is strictly dependent on the recovery time of the cellular membrane after activation. Once an action potential is initiated at one point in the cell body, its propagation along the axon occurs through a chain of depolarization-repolarization membrane processes. An action potential only occurs in a limited area of the cell membrane, but the changes in membrane potential at the edges of this area are enough to initiate another action potential in a neighboring space, which then give rise to another action potential further down the axon, a process which results in the propagation of the action potential. Each single point of the axon and its neighborhood experience initiation of an action potential followed by a period of recovery during which Na^+ channels are in their inactive state, and the axon segment is refractory. Given the spatially local area of how we defined nodes along the axon and axonal arborization (see above sections), we assumed that every vertex in our tree graph model of each neuron could be associated with a locally active-refractory area of membrane along the axon, and that a locally-defined refractory period could be calculated for it.

Our estimation of the refractory period at individual vertices along the axon was based on very recent findings about the biophysical and dynamical properties of basket cells. In the work by Hu and colleagues[1] these authors carried out confocally visualized sub-cellular patch-clamp recordings in rat hippocampal slices at different sites in the soma and axons of basket cells. Their experimental measurements demonstrated a supercritical density of sodium ion (Na^+) channels with a non-uniform density profile along the axonal tree. In particular, sub-cellular mapping revealed a stepwise increase of Na^+ conductance density from the soma ($\rho_{Na} = 2.6$ channels μm^{-2}) to the proximal axon ($\rho_{Na} = 25$ channels μm^{-2} at 50 μm distance from the soma), followed by an additional gradual increase in the distal axon ($\rho_{Na} = 46.1$ channels μm^{-2} at 315 μm distance from the soma). High Na^+ channel density is normally required for action potential generation; in typical neuron axons Na^+ channels are typically concentrated at the axon initial segment where action potential generation occurs. Based on this detailed work however, Na^+ channel distribution differed somewhat in basket cell axons. This supercritical, gradual increase in the density of sodium channels was interpreted as ensuring high reliability and fast propagation of neural signaling in unmyelinated small axons[1]. Additionally, analysis of gating kinetics and voltage-dependence of activation and inactivation revealed that Na^+ channels in basket cell axons differed from somatic channels in their functional properties. In particular, the inactivation time constant was 2.4-fold faster for axonal than for somatic Na^+ channels, thus implying locally reduced refractory periods in the axon with respect to the soma.

Given this information, we decided to approximate the decreasing refractory period from soma to synaptic terminals with a curve whose descending slope was inferred from the actual measurements of channel density variation. Specifically, a lowest and highest bound were defined for the refractory period. The lowest bound was set to 1 ms, which is about the absolute lower limit for any neuron due to fundamental limitations associated with the kinetics of Na^+ channels. The maximum value for R_j was set to 2.5 ms, which we justify by three specific arguments: first, 2.5 ms represents a reasonable value as the average of the generally accepted range (0 - 5 ms) associated with neocortical neurons[2–5]; second, the work on demyelinated axons and the supercritical Na^+ channel density proposed in[1] implies that higher values would not be physically appropriate; finally, a change of refractory period from 1 ms to 2.5 ms is in agreement with the 2.4-fold increase of activation time for Na^+ channels suggested by the experiments in[1]. We thus determined the refractory period for any output vertex v_j of individual axon branches as a function of the distance of the vertex from the soma. Specifically, R_j values steeply decrease from 2.5 ms to $\sim$ 1.7 ms in the first 50 μm distance from the cell main body; it then further gradually reduces at the distal site of the axon until reaching a constant refractory period of 1 ms at a threshold length of 315 μm (Figure 1).

The function used to estimate the varying refractory period along individual branches was constructed from considerations of the experimental curve obtained for the Na^+ channel density variation along basket cell axons from the initial segment to distal parts of the axons (315 um) in[1] (see Figure 1 in[1]). The channel density steeply increased from the soma to the proximal axon out to about 50 μm from a density of 2.6 channels μm^2 to a value of about 25 channels μm^2. We empirically estimated the slope of the curve describing the profile of channel density change within this first section of the axon by taking the slope of the straight line connecting these two points in that data. Beyond about 50 μm the channel density increased less steeply from a value of 25 channels μm^2 to approximately 46.1 channels μm^2 at

315 μm from the soma. We similarly linearly estimated the changing channel density in this second section of the axon by calculating the slope of the line connecting these two points.

We then built the function approximating the gradual decrease of the refractory period along individual branches as a function of the distance from the soma or axon initial segment.

Since no information was reported for distances from the soma larger than 315 μm, and given the gradual decrease in the slope relative to the channel density curve, we assumed that by 315 μm^2 the refractory period nearly reached saturation at a minimal value of 1 ms, and assumed that beyond that distance any further changes in the refractory period were negligible. Moreover, observing that for larger distances the average diameter of axonal arborizations could be assumed to have no relevant variation, we found it to be a reasonable assumption since the geometry does affect the recovery properties of the membrane[6]. We also assumed that the refractory period varies along individual branches from the minimal value of 1 ms to the maximum of 2.5 ms as the inverse of the channel density profile, since as discussed above there is an inverse relationship between the refractory period and channel density. In an ideal scenario empirical data for the refractory period would exist along the continuum of axonal branches. Since this is not the case, we made assumptions that reflect the most current and detailed data available about the refractory period of Basket cell neurons. Importantly, we modeled the refractory period before and independent of calculations of the refraction ratio. In other words, the refractory period model was not adjusted to achieve an optimal ratio.

Trade-Off Conservation at The Synapses

In order to investigate how the dynamic properties vary throughout the axon hierarchy, we calculated the evolution of the ratio along individual axonal paths. We computed the refraction ratio for sequential pairs of vertices where each pair includes the root vertex v_R as source vertex and one of the $Q-1$ branching points v_B as output vertex, where each branching vertex is selected one at a time descending along the axonal branch (or path $p(v_R, v_{T_s})$) to the synaptic terminal (Figure 2 A). In Figure 2B we report the evolving refraction ratio for all branches of a single basket cell (coded as different colors for each branch; Fig.2B). When evaluated this way, there is a converging trend of the refraction ratio towards optimality as it approaches the terminal output vertices. The inset in Figure 2B shows that a high percentage of the total number of branches in the cell have high values of refraction ratio early in the tree, which then converge quickly to near-optimal values as the terminal synaptic vertices for each branch are approached (*c.f.* Figure 2 in the main text).

Figure 3A shows the distribution towards optimal convergence of the refraction ratio for a representative basket cell as a function of the branching order at which convergence towards the optimal range occurs. The branching order corresponds to depth of a selected branching vertex on the axonal path $p(v_R, v_{T_s})$. The vertex depth is here defined by the number of bifurcations that separate that specific branching vertex from the root vertex. For this analysis, we arbitrarily fixed the range $0.5 \leq \Lambda_{ij} \leq 1.5$ as optimality range of convergence. The histogram shows what is the smallest branching order in the axon hierarchy at which the refraction ratio converges. Figure 3B shows the same metric combined for our whole dataset of 57 neurons. More specifically, the histogram shows the number of neurons whose maximum number of branches have ratio converging to optimality as a function of the first branching at which convergence occurs. To build this histogram, we first selected, for each neuron in the data set, the branches whose ratio at synapses falls within an optimal range (here arbitrarily set to $0.5 \leq \Lambda_{ij} \leq 1.5$); then, for each branch we determined the branching order in the hierarchy at which convergence first occurs, as described for data in Figure 3A; finally, for each of the 57 cells, we calculated the order of bifurcation that maximizes the number of converging branches. We obtained 57 different orders, one for each neuron, and used them to build the here reported distribution.

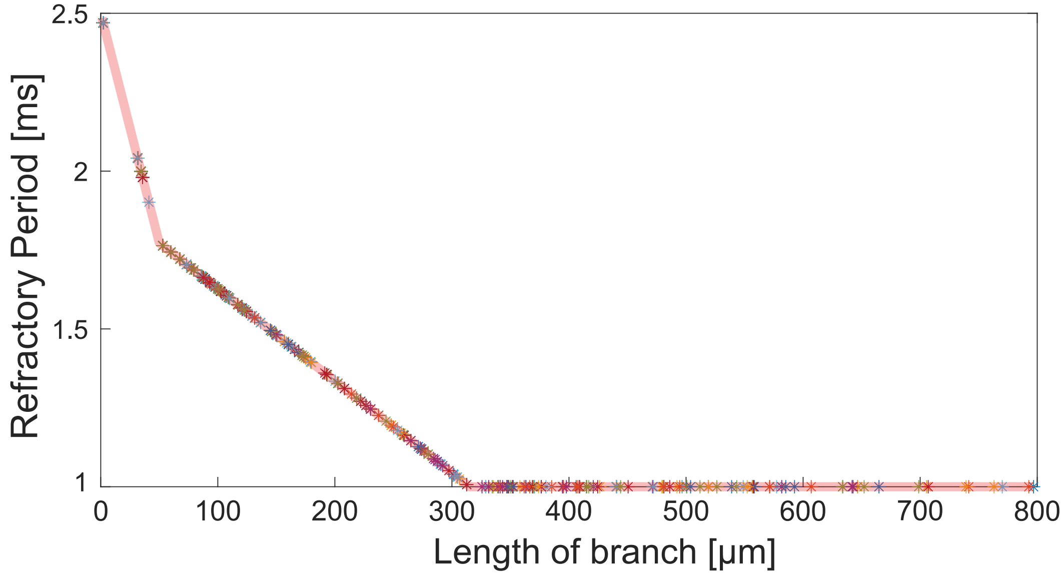

Figure 1. Estimation of the refractory period for an individual basket cell. R_j is plotted as a function of the path length. Its values steeply decrease from 2.5 ms to $\sim$ 1.7 ms in the first 50 μm distance from the cell main body; then, R_j gradually reduces at the distal site of the axon until reaching a constant refractory period of 1 ms at a threshold length of 315 μm.

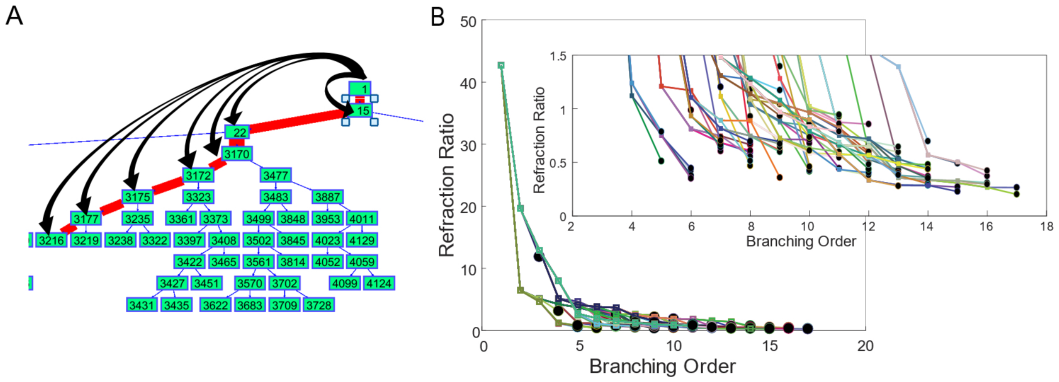

Figure 2. Convergence of the refraction ratio towards an optimal range for one representative basket cell neuron. (A) Schematic representation of the different pairs of vertices for which the evolving ratio was computed, starting from the root vertex v_R at the soma and ending at the synaptic terminals v_{T_s} for all paths $p(v_R, v_{T_s})$. (B) Evolving signaling ratio for all paths in the axon arbors of the same cell. Different colors represent different branches. The x-axes shows the order of bifurcation (depth of the tree) in the hierarchical structure from the origin to the terminal vertex. The y-axes shows the value of the computed refraction ratios. Inset: zoomed in view of the same data. We assumed an optimal refraction ratio range to be within 0.5 and 1.5 for this analysis.

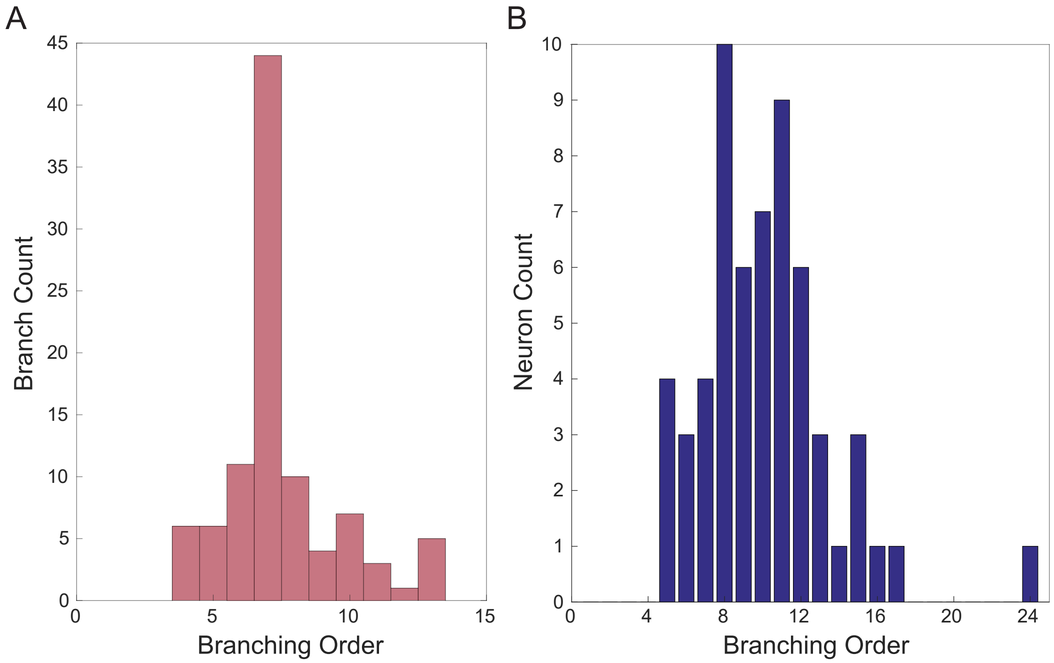

Figure 3. Branching order counts at which an optimal ratio is achieved. (A) Histogram showing the distribution of branching orders (depth in the tree-network) for a single neuron at which an optimal ratio is reached. We assumed the same optimal ratio 0.5 and 1.5 as in the rest of the analysis. (B) Data showing the results of the same analyis as for the representative neuron in panel A, but plotting for each neuron only the branch order that displayed the maximum number of axonal branches that achieved the optimal ratio at that particular branch order (see text).

References

1. Hu, H. & P., J. A supercritical density of Na^+ channels ensures fast signaling in GABAergic interneuron axons. *Nat. Neurosci.* **17**, 686–693 (2014).
2. Chen, N., Yu, J., Qian, H., Ge, R. & Wang, J.-H. Axons Amplify Somatic Incomplete Spikes into Uniform Amplitudes in Mouse Cortical Pyramidal Neurons. *PLoS ONE* **5**, 1–10 (2010).
3. Khaliq, Z. M. & Raman, I. M. Axonal Propagation of Simple and Complex Spikes in Cerebellar Purkinje Neurons. *J. Neurosci.* **25**, 454–463 (2005).
4. Berry, M. J. & Meister, M. Refractoriness and Neural Precision. *J. Neurosci.* **18**, 2200–2211 (1998).
5. Shu, Y., Duque, A., Yu, Y., Haider, B. & McCormick, D. A. Properties of Action-Potential Initiation in Neocortical Pyramidal Cells: Evidence From Whole Cell Axon Recordings. *J. Neurophysiol.* **97**, 746–760 (2007).
6. Paintal, A. The influence of diameter of medullated nerve fibres of cats on the rising and falling phases of the spike and its recovery. *J. Physiol.* **184**, 791–811 (1966).
